# Genuine modified Bernstein–Durrmeyer operators

**DOI:** 10.1186/s13660-018-1693-z

**Published:** 2018-05-03

**Authors:** Syed Abdul Mohiuddine, Tuncer Acar, Mohammed A. Alghamdi

**Affiliations:** 1 Operator Theory and Applications Research Group, Department of Mathematics, Faculty of Science, King Abdulaziz University, Jeddah, Saudi Arabia; 2 Department of Mathematics, Faculty of Science, Selcuk University, Selcuklu, Konya, Turkey

**Keywords:** 41A25, 41A35, 41A36, Genuine Bernstein–Durrmeyer operators, Rate of convergence, Quantitative Voronovskaya theorem, Gruss–Voronovskaya theorem

## Abstract

The present paper deals with genuine Bernstein–Durrmeyer operators which preserve some certain functions. The rate of convergence of new operators via a Peetre $\mathcal{K}$-functional and corresponding modulus of smoothness, quantitative Voronovskaya type theorem and Grüss–Voronovskaya type theorem in quantitative mean are discussed. Finally, the graphic for new operators with special cases and for some values of *n* is also presented.

## Introduction

Bernstein polynomials have a crucial role in the theory of approximation by positive linear operators due to their simple and useful structure. In their long historical progress, different types of research were dedicated to improving the rate of convergence and decreasing the error of the approximation. On the other hand, Bernstein polynomials have been transferred to a space of functions being Lebesgue integrable and Riemann integrable.

In a recent paper [1], Cárdenas-Morales et al. considered a new construction of Bernstein polynomials for $f\in C [ 0,1 ] $,

1.1 $$ B_{n}^{\tau } ( f;x ) =\sum_{k=0}^{n} \bigl( f\circ \tau^{-1} \bigr) \biggl( \frac{k}{n} \biggr) p_{n,k}^{\tau } ( x ) , $$

where $p_{n,k}^{\tau } ( x ) =\binom{n}{k} ( \tau ( x ) ) ^{k} ( 1-\tau ( x ) ) ^{n-k}$, $x\in [ 0,1 ] $, *τ* is a continuous infinite times differentiable function satisfying the condition $\tau ( 1 ) =1$, $\tau ( 0 ) =0$, and $\tau^{\prime } ( x ) >0$ for $x\in [ 0,1 ] $. By this construction, the Korovkin set is generalized from $\{ 1,e_{1},e_{2} \} $ to $\{ 1, \tau ,\tau^{2} \} $ and it was shown that the $B_{n}^{\tau }$ present a better degree of approximation depending on *τ*. Inspired by this idea, many researchers have performed studied in this direction. In some of these studies generalized Szász type operators depending on *τ* were mentioned in [2] and further properties in [3], Bernstein–Kantorovich operators in [4] (also see [5]), Szász–Durrmeyer operators in [6], Gamma operators in [7]. Very recently, Acar et al. [8] have introduced Durrmeyer modifications of the operators (1.1):

1.2 $$ D_{n}^{\tau } ( f;x ) = ( n+1 ) \sum _{k=0}^{n}p_{n,k} ^{\tau } ( x ) \int_{0}^{1} \bigl( f\circ \tau^{-1} \bigr) ( t ) p_{n,k} ( t )\,dt, $$

where

$$ p_{n,k} ( t ) =\binom{n}{k}x^{k} ( 1-x ) ^{n-k}, \quad x \in [0,1 ] . $$

The operators defined in (1.2) are linear and positive. In case of $\tau ( x ) =x$, the operators in (1.2) reduce to well-known Bernstein–Durrmeyer operators introduced by Durrmeyer [9] and they have been intensively studied by Derriennic [10]. The rate of convergence and pointwise convergence of the operators in (1.2) via a quantitative Voronovskaya type theorem were discussed, and also the flexibility and sensitivity of new operators were presented with graphics and numerical results.

Other useful modifications of positive linear operators are genuine types in approximation theory. These modifications for Bernstein–Durrmeyer operators were first considered by Chen [11] and, a year later, by Goodman and Sharma [12]. Since then, many researchers have studied in this direction, among others we have the authors’ work on genuine Bernstein–Durrmeyer operators, we can mention some important work such as Gonska et al. [13], Parvanov and Popov [14], etc. Note that Bernstein–Durrmeyer operators preserve only the constant functions, but with the modifications mentioned, linear functions are preserved which allows us to present a better rate of convergence.

The aim of this paper is to introduce the genuine Bernstein–Durrmeyer operators which preserve the function *τ* and investigate the rate of convergence of our operators in terms of second-order modulus of continuity and the Ditzian–Totik modulus of continuity. To describe the pointwise convergence of the operators, we prove a quantitative Vorononskaya type theorem in terms of the least concave majorant of the classical modulus of continuity. This quantitative Voronovskaya theorem tells us the rate of pointwise convergence and an upper bound for the error of the approximation. For the most recent papers on quantitative Voronovskaya theorems, we refer to [15–18].

## Construction of the operators and auxiliary lemmas

In the present paper, we construct a genuine type modification of the operators in (1.2) which preserve the function *τ*, defined as

2.1 $$\begin{aligned} G_{n}^{\tau } ( f;x ) &= ( n-1 ) \sum _{k=1}^{n-1}p _{n,k}^{\tau } ( x ) \int_{0}^{1} \bigl( f\circ \tau^{-1} \bigr) ( t ) p_{n-2,k-1} ( t )\,dt \\ &\quad {}+p_{n,0}^{\tau } ( x ) \bigl( f\circ \tau^{-1} \bigr) ( 0 ) +p_{n,n}^{\tau } ( x ) \bigl( f\circ \tau ^{-1} \bigr) ( 1 ) \end{aligned}$$

and we call these operators genuine modified Bernstein–Durrmeyer operators. Clearly, the operators defined in (2.1) are linear and positive. Further, in the case of $\tau ( x ) =x$, the operators in (2.1) reduce to the following operators introduced in [11, 12]:

$$\begin{aligned} U_{n} ( f;x ) = ( n-1 ) \sum_{k=1}^{n-1}p_{n,k} ( x ) \int_{0}^{1}f ( t ) p_{n-2,k-1} ( t )\,dt +p_{n,0} ( x ) f ( 0 ) +p_{n,n} ( x ) f ( 1 ) . \end{aligned}$$

To prove our main results, we need moments and central moments of our new operators. The proofs of the following two lemmas will not be presented since they can be obtained by the operators $U_{n}$ (see [13, Proposition 2.5]).

### Lemma 1

*Let* $e_{i}^{\tau }=\tau^{i}$, $i=0,1,2,\ldots $ . *Then we have*

2.2 $$\begin{aligned} &G_{n}^{\tau }e_{0}^{\tau } = 1, \\ &G_{n}^{\tau }e_{1}^{\tau } = \tau , \\ &G_{n}^{\tau }e_{2}^{\tau } = \tau^{2}+\frac{2\tau ( 1-\tau )}{n+1}, \\ &G_{n}^{\tau }e_{3}^{\tau } = \frac{ ( n-1 ) ( n-2 ) \tau^{3}+6 ( n-1 ) \tau^{2}+6\tau }{ ( n+1 ) ( n+2 ) }, \\ &G_{n}^{\tau }e_{4}^{\tau } = \frac{ ( n-1 ) ( n-2 ) ( n-3 ) \tau^{4}}{ ( n+1 ) ( n+2 ) ( n+3 ) }+\frac{ (12n^{2}-36n+24 ) \tau^{3}}{ ( n+1 ) ( n+2 ) ( n+3 ) } \\ &\hphantom{G_{n}^{\tau }e_{4}^{\tau } = }{}+\frac{36 ( n-1 ) \tau^{2}}{ ( n+1 ) ( n+2 ) ( n+3 ) }+\frac{24\tau }{ ( n+1 ) ( n+2 ) ( n+3 ) }. \end{aligned}$$

### Lemma 2

*For* $m,n\in \mathbb{N}$ *and* $x\in [ 0,1 ] $, *let the central moment operator be given by*

$$ M_{n,m}^{\tau } ( x ) =G_{n}^{\tau } \bigl( \bigl( \tau ( t ) -\tau ( x ) \bigr) ^{m};x \bigr) . $$

*Then we have*

2.3 $$\begin{aligned} &M_{n,0}^{\tau } ( x ) =1, \\ &M_{n,1}^{\tau } ( x ) =0, \end{aligned}$$

2.4 $$\begin{aligned} &M_{n,2}^{\tau } ( x ) =\frac{2\tau ( x ) ( 1- \tau ( x ) ) }{n+1}, \end{aligned}$$

2.5 $$\begin{aligned} &M_{n,3}^{\tau } ( x ) =\frac{6\tau ( x ) ( 1- \tau ( x ) ) ( 1-2\tau ( x ) ) }{ (n+1 ) ( n+2 ) }, \\ &M_{n,4}^{\tau } ( x ) =\frac{12 (1-\tau ( x ) ) ^{2}\tau^{2} ( x ) ( n-7 ) +24 ( 1- \tau ( x ) ) \tau ( x ) }{ ( n+1 ) ( n+2 ) ( n+3 ) }. \end{aligned}$$

### Lemma 3

*For* $f\in C [ 0,1 ] $, *we have* $\Vert G_{n}^{\tau }\Vert \leq \Vert f\Vert $, *where* $\Vert \cdot \Vert $ *is the sup*-*norm on* $[ 0,1 ]$.

### Proof

From (2.1) and (2.2), one gets

$$ \bigl\Vert G_{n}^{\tau }\bigr\Vert \leq \bigl\Vert f\circ \tau^{-1}\bigr\Vert G _{n}^{\tau }e_{0}^{\tau }= \Vert f\Vert . $$

 □

### Lemma 4

*For* $n\in \mathbb{N}$ *and* $x\in [ 0,1 ] $, *one has*

2.6 $$ \frac{M_{n,4}^{\tau } ( x ) }{M_{n,2}^{\tau } ( x ) }\leq \frac{6}{ ( n+3 ) }. $$

### Proof

Using (2.5) and (2.4), we can write

$$\begin{aligned} \begin{aligned} \frac{M_{n,4}^{\tau } ( x ) }{M_{n,2}^{\tau } ( x ) } &=\frac{12\tau^{2} ( x ) ( 1-\tau ( x ) ) ^{2} ( n-7 ) +24\tau ( x ) ( 1-\tau ( x ) ) }{ ( n+1 ) ( n+2 ) ( n+3 ) }\frac{n+1}{2\tau ( x ) ( 1-\tau ( x ) ) } \\ &=\frac{2\tau ( x ) ( 1-\tau ( x ) ) [ 6\tau ( x ) ( 1-\tau ( x ) ) ( n-7 ) +12 ] }{ ( n+2 ) ( n+3 ) }\frac{1}{2 ( 1-\tau ( x ) ) \tau ( x ) } \\ &=\frac{6 ( 1-\tau ( x ) ) \tau ( x ) ( n-7 ) +12}{ ( n+2 ) ( n+3 ) }. \end{aligned} \end{aligned}$$

Since $0\leq \tau ( x ) \leq 1$, $\tau ( x ) ( 1-\tau ( x ) ) \leq 1$, we have

$$ \frac{M_{n,4}^{\tau } ( x ) }{M_{n,2}^{\tau } ( x ) }\leq \frac{6 ( n-7 ) +12}{ ( n+2 ) ( n+3 ) }=\frac{6n-30}{ ( n+2 ) ( n+3 ) }\leq \frac{6}{ ( n+3 ) }. $$

 □

## Direct theorems

In this section, first we obtain a direct result for the operators $G_{n}^{\tau }$. Let us first recall some auxiliary functions. The Peetre’s *K*-functional [19] is defined by

$$ K ( f,\delta ) =\inf_{g\in W^{2}} \bigl\{ \Vert f-g\Vert + \delta \Vert g\Vert _{W^{2}} \bigr\} , $$

where

$$ \Vert g\Vert _{W^{2}}=\Vert g\Vert +\bigl\Vert g ^{\prime } \bigr\Vert +\bigl\Vert g^{\prime \prime }\bigr\Vert $$

and

$$ W^{2}= \bigl\{ g\in C [ 0,1 ] :g^{\prime },g^{\prime \prime }\in C [ 0,1 ] \bigr\} . $$

Also, as is known from Proposition 3.4.1 of [20] there is a constant $C>0$ such that

3.1 $$ K ( f,\delta ) \leq C \bigl( \omega_{2} ( f,\sqrt{ \delta } ) +\min \{ 1,\delta \} \Vert f\Vert \bigr) $$

for all $f\in C [ 0,1 ] $ and $x\in [ 0,1 ] $, where

$$ \omega_{2} ( f,\delta ) = \sup_{\vert h\vert < \delta } \sup _{x,x+2h\in [ 0,1 ] }\bigl\vert f ( x+2h ) -2f ( x+h ) +f ( x ) \bigr\vert $$

is the second-order modulus of continuity of *f*. The usual modulus of continuity of $f\in C [ 0,1 ] $ is defined by

$$ \omega ( f,\delta ) = \sup_{\vert h\vert < \delta } \sup_{x,x+h\in [ 0,1 ] } \bigl\vert f ( x+h ) -f ( x ) \bigr\vert . $$

### Theorem 1

*Let* $f\in C [ 0,1 ] $, $x\in [ 0,1 ] $ *and* $\inf_{x\in [ 0,1 ] }\tau^{\prime } ( x ) \geq a$, *where* $a\in \mathbb{R}^{+}$. *There exists a positive constant* $C_{1}$ *independent of* *f* *and* *n* *such that the inequality*

$$ \bigl\vert G_{n}^{\tau } ( f;x ) -f ( x ) \bigr\vert \leq C_{1} \biggl[ \omega_{2} \biggl( f,\frac{\varphi_{n} ( x ) }{ ( n+1 ) ^{1/2}} \biggr) +\min \biggl\{ 1,\frac{\varphi_{n} ^{2} ( x ) }{n+1} \biggr\} \Vert f\Vert \biggr] $$

*holds*, *where*

$$ \varphi_{n} ( x ) =\sqrt{ \bigl( 1-\tau ( x ) \bigr) \tau ( x ) }\quad \bigl(x\in [ 0,1 ] \bigr). $$

### Proof

By Taylor’s formula, for $g\in W^{2}$, we can write

3.2 $$\begin{aligned} g ( t ) =& \bigl( g\circ \tau^{-1} \bigr) \tau ( x ) + \bigl( \tau ( t ) -\tau ( x ) \bigr) D \bigl( g \circ \tau^{-1} \bigr) \tau ( x ) \\ &+ \int_{\tau ( x ) }^{\tau ( t ) }D^{2} \bigl( g \circ \tau^{-1} \bigr) ( u ) \bigl( \tau ( t ) -u \bigr)\,du. \end{aligned}$$

On the other hand, since

$$\begin{aligned}& \int_{\tau ( x ) }^{\tau ( t ) } \bigl( \tau ( t ) -u \bigr) ^{2}D^{2} \bigl( g\circ \tau^{-1} \bigr) ( u )\,du \\& \quad = \int_{x}^{t} \bigl( \tau ( t ) -\tau ( y ) \bigr) D^{2} \bigl( g\circ \tau^{-1} \bigr) \tau ( y ) \tau^{\prime } ( y )\,dy \end{aligned}$$

and

3.3 $$ D^{2} \bigl( g\circ \tau^{-1} \bigr) \tau ( y ) = \frac{1}{ \tau^{\prime } ( y ) } \biggl[ \frac{g^{\prime \prime } ( y ) \tau^{\prime } ( y ) -g^{\prime } ( y ) \tau^{\prime \prime } ( y ) }{ ( \tau^{\prime } ( y ) ) ^{2}} \biggr] , $$

we can write

3.4 $$\begin{aligned} & \int_{\tau ( x ) }^{\tau ( t ) } \bigl( \tau ( t ) -u \bigr) D^{2} \bigl( g\circ \tau^{-1} \bigr) ( u )\,du \\ & \quad = \int_{x}^{t} \bigl( \tau ( t ) -\tau ( y ) \bigr) \biggl[ \frac{g^{\prime \prime } ( y ) \tau^{\prime } ( y ) -g^{\prime } ( y ) \tau^{\prime \prime } ( y ) }{ ( \tau^{\prime } ( y ) ) ^{2}} \biggr]\,dy \\ & \quad = \int_{\tau ( x ) }^{\tau ( t ) } \bigl( \tau ( t ) -u \bigr) \frac{g^{\prime \prime } ( \tau^{-1} ( u ) ) }{ ( \tau^{\prime } ( \tau^{-1} ( u ) ) ) ^{2}}\,du \\ & \quad \quad {}- \int_{\tau ( x ) }^{\tau ( t ) } \bigl( \tau ( t ) -u \bigr) \frac{g^{\prime } ( \tau^{-1} ( u ) ) \tau^{\prime \prime } ( \tau^{-1} ( u ) ) }{ ( \tau^{\prime } ( \tau^{-1} ( u ) ) ) ^{3}}\,du. \end{aligned}$$

If the operators $G_{n}^{\tau }$ are applied to both sides of the equality (3.2) and the equality (3.4) is considered with the assumption $\inf_{x\in [ 0,1 ] }\tau^{\prime } ( x ) \geq a$, one obtains

3.5 $$\begin{aligned} \bigl\vert G_{n}^{\tau } ( g;x ) -g ( x ) \bigr\vert & \leq G_{n}^{\tau } \biggl( \biggl\vert \int_{\tau ( x ) } ^{\tau ( t ) } \bigl( \tau ( t ) -u \bigr) D^{2} \bigl( g\circ \tau^{-1} \bigr) ( u )\,du\biggr\vert ;x \biggr) \\ &\leq G_{n}^{\tau } \biggl( \biggl\vert \int_{\tau ( x ) } ^{\tau ( t ) }\frac{g^{\prime \prime } ( \tau^{-1} ( u ) ) }{ ( \tau^{\prime } ( \tau^{-1} ( u ) ) ) ^{2}} \bigl( \tau ( t ) -u \bigr)\,du\biggr\vert ;x \biggr) \\ &\quad {}+G_{n}^{\tau } \biggl( \biggl\vert \int_{\tau ( x ) }^{ \tau ( t ) } \frac{g^{\prime } ( \tau^{-1} ( u ) ) \tau^{\prime \prime } ( \tau^{-1} ( u ) ) }{ ( \tau^{\prime } ( \tau^{-1} ( u ) ) ) ^{3}} \bigl( \tau ( t ) -u \bigr)\,du\biggr\vert ;x \biggr) \\ &\leq G_{n}^{\tau } \bigl( \bigl( \tau ( t ) -\tau ( x ) \bigr) ^{2};x \bigr) \biggl[ \frac{\Vert g^{\prime \prime }\Vert }{a^{2}}+\frac{\Vert g^{\prime }\Vert \Vert \tau^{\prime \prime }\Vert }{a^{3}} \biggr] \\ &\leq \frac{2\varphi_{n}^{2} ( x ) }{n+1} \biggl[ \frac{\Vert g ^{\prime \prime }\Vert }{a^{2}}+\frac{\Vert g^{\prime }\Vert \Vert \tau^{\prime \prime }\Vert }{a^{3}} \biggr]. \end{aligned}$$

For any $f\in C [ 0,1 ] $ and $g\in W^{2}$, using (3.5), we have

$$\begin{aligned} \bigl\vert G_{n}^{\tau } ( f;x ) -f ( x ) \bigr\vert & \leq\bigl\vert g ( x ) -f ( x ) \bigr\vert + \bigl\vert G_{n}^{\tau } ( f-g;x ) \bigr\vert +\bigl\vert G _{n}^{\tau } ( g;x ) -g ( x ) \bigr\vert \\ &\leq 2\Vert f-g\Vert +\frac{2\varphi_{n}^{2} ( x ) }{n+1} \biggl[ \frac{\Vert g^{\prime \prime }\Vert }{a ^{2}}+ \frac{\Vert g^{\prime }\Vert \Vert \tau^{\prime \prime }\Vert }{a^{3}} \biggr] \\ &\leq 2\Vert f-g\Vert +\frac{2\varphi_{n}^{2} ( x ) }{n+1} \biggl[ \frac{\Vert g^{\prime \prime }\Vert }{a ^{2}}+ \frac{\Vert g^{\prime }\Vert \Vert \tau^{\prime \prime }\Vert }{a^{3}}+\Vert g\Vert \biggr] . \end{aligned}$$

If we choose $C:=\max \{ 2,\frac{2}{a^{2}},\frac{2\Vert \tau^{ \prime \prime }\Vert }{a^{3}} \} $, then we can write

$$ \bigl\vert G_{n}^{\tau } ( f;x ) -f ( x ) \bigr\vert \leq 2 \Vert f-g\Vert +C\frac{\varphi_{n}^{2} ( x ) }{n+1} \bigl[ \bigl\Vert g^{\prime \prime }\bigr\Vert +\bigl\Vert g ^{\prime }\bigr\Vert +\Vert g\Vert \bigr] . $$

Taking the infimum on the above inequality over all $g\in W^{2}$, one gets

$$ \bigl\vert G_{n}^{\tau } ( f;x ) -f ( x ) \bigr\vert \leq CK \biggl( f,\frac{\varphi_{n}^{2} ( x ) }{n+1} \biggr) . $$

Using Eq. (3.1), we get

$$ \bigl\vert G_{n}^{\tau } ( f;x ) -f ( x ) \bigr\vert \leq C_{1} \biggl[ \omega_{2} \biggl( f,\frac{\varphi_{n} ( x ) }{ ( n+1 ) ^{1/2}} \biggr) +\min \biggl\{ 1,\frac{\varphi_{n} ^{2} ( x ) }{n+1} \biggr\} \Vert f\Vert \biggr] , $$

which proves the theorem completely. □

### Theorem 2

*Let* $f\in C^{1} [ 0,1 ] $. *Then*, *for every* $x\in [ 0,1 ] $, *the inequality*

$$ \bigl\vert G_{n}^{\tau } ( f;x ) -f ( x ) \bigr\vert \leq 2 \varphi_{n} ( x ) \omega \bigl( D \bigl( f\circ \tau^{-1} \bigr) ,\varphi_{n} ( x ) \bigr) $$

*holds*.

### Proof

For any $x\in [ 0,1 ] $ and $t\in [ 0,1 ] $, we have

$$\begin{aligned} f ( t ) &= \bigl( f\circ \tau^{-1} \bigr) \tau ( x ) +D \bigl( f\circ \tau^{-1} \bigr) \tau ( x ) \bigl( \tau ( t ) -\tau ( x ) \bigr) \\ &\quad {}+ \int_{\tau ( x ) }^{\tau ( t ) } \bigl[ D \bigl( f\circ \tau^{-1} \bigr) ( u ) -D \bigl( f\circ \tau^{-1} \bigr) \bigl( \tau ( x ) \bigr) \bigr]\,du. \end{aligned}$$

Applying the operators $G_{n}^{\tau }$ to both sides of the above equality, we obtain

$$\begin{aligned} G_{n}^{\tau } \bigl( f ( t ) -f ( x ) ;x \bigr) &=D \bigl( f \circ \tau^{-1} \bigr) \tau ( x ) G_{n}^{\tau } \bigl( \bigl( \tau ( t ) -\tau ( x ) \bigr) ;x \bigr) \\ &\quad {}+G_{n}^{\tau } \biggl( \int_{\tau ( x ) }^{\tau ( t ) } \bigl[ D \bigl( f\circ \tau^{-1} \bigr) ( u ) -D \bigl( f\circ \tau^{-1} \bigr) \bigl( \tau ( x ) \bigr) \bigr]\,du;x \biggr) . \end{aligned}$$

On the other hand, since the usual modulus of continuity has the property

$$ \bigl\vert f ( u ) -f ( x ) \bigr\vert \leq \omega ( f,\delta ) \biggl( 1+ \frac{\vert u-x\vert }{ \delta } \biggr)\quad (\delta >0), $$

we can write

$$\begin{aligned}& \biggl\vert \int_{\tau ( x ) }^{\tau ( t ) } \bigl[ D \bigl( f\circ \tau^{-1} \bigr) ( u ) -D \bigl( f\circ \tau^{-1} \bigr) \bigl( \tau ( x ) \bigr) \bigr]\,du\biggr\vert \\& \quad \leq \omega \bigl( D \bigl( f\circ \tau^{-1} \bigr) ,\delta \bigr) \biggl( \frac{ ( \tau ( t ) -\tau ( x ) ) ^{2}}{\delta }+\bigl\vert \tau ( t ) -\tau ( x ) \bigr\vert \biggr) . \end{aligned}$$

Thus, we obtain

$$\begin{aligned} \begin{aligned} \bigl\vert G_{n}^{\tau } ( f;x ) -f ( x ) \bigr\vert & \leq \bigl\vert D \bigl( f\circ \tau^{-1} \bigr) \tau ( x ) \bigr\vert \bigl\vert M_{n,1}^{\tau } ( x ) \bigr\vert + \omega \bigl( D \bigl( f\circ \tau^{-1} \bigr) ,\delta \bigr) \\ &\quad {}+\omega \bigl( D \bigl( f\circ \tau^{-1} \bigr) ,\delta \bigr) \biggl\{ \frac{1}{\delta }M_{n,2}^{\tau } ( x ) +G_{n}^{ \tau } \bigl( \bigl\vert \tau ( t ) -\tau ( x ) \bigr\vert ;x \bigr) \biggr\} . \end{aligned} \end{aligned}$$

Applying the Cauchy–Schwarz inequality, one gets

$$ \bigl\vert G_{n}^{\tau } ( f;x ) -f ( x ) \bigr\vert \leq \omega \bigl( D \bigl( f\circ \tau^{-1} \bigr) ,\delta \bigr) \sqrt {M _{n,2}^{\tau } ( x ) } \biggl\{ \frac{1}{\delta }\sqrt {M _{n,2}^{\tau } ( x ) }+1 \biggr\} . $$

If we choose $\delta =\sqrt{M_{n,2}^{\tau } ( x ) }$, then we find

$$\begin{aligned} \bigl\vert G_{n}^{\tau } ( f;x ) -f ( x ) \bigr\vert &\leq 2 \omega \Bigl( D \bigl( f\circ \tau^{-1} \bigr) ,\sqrt {M_{n,2} ^{\tau } ( x ) } \Bigr) \sqrt {M_{n,2}^{\tau } ( x ) } \\ &=2\varphi_{n} ( x ) \omega \bigl(D \bigl( f\circ \tau^{-1} \bigr) ,\varphi_{n} ( x ) \bigr), \end{aligned}$$

which is the desired result. □

## Voronovskaya and Grüss–Voronovskaya type theorems

The next result is a quantitative Voronovskaya type theorem which describes the rate of pointwise convergence of the operators $G_{n}^{\tau }$. In [21], Gonska et al. presented the following theorem which is a quantitative Voronovskaya type theorem for a general sequence of positive linear operators acting on compact intervals.

### Theorem 3

([21]) *Suppose* $L_{n}:C [ 0,1 ] \rightarrow C [ 0,1 ] $ ($n\geq 1$) *is a linear positive operators satisfying* $L_{n}e_{0}=e_{0}$. *If* $x\in [ 0,1 ] $ *and* $f\in C^{2} [ 0,1 ] $, *then*

$$\begin{aligned}& \biggl\vert L_{n} ( f;x ) -f ( x ) -f^{\prime } ( x ) L_{n} \bigl( ( e_{1}-x ) ;x \bigr) - \frac{1}{2}f^{\prime \prime } ( x ) L_{n} \bigl( ( e_{1}-x ) ^{2};x \bigr) \biggr\vert \\& \quad \leq \frac{1}{2}L_{n} \bigl( ( e_{1}-x ) ^{2};x \bigr) \tilde{\omega } \biggl( f^{\prime \prime }, \frac{1}{3}\sqrt{\frac{L_{n} ( ( e_{1}-x ) ^{4};x ) }{L_{n} ( ( e_{1}-x ) ^{2};x ) }} \biggr) , \end{aligned}$$

*where* $\tilde{\omega } ( f^{\prime \prime },\cdot ) $ *denotes the least concave majorant of* $\omega (f,\cdot )$ *given by*

$$ \tilde{\omega } ( f,\varepsilon ) = \textstyle\begin{cases} \sup\limits_{\substack{0\leq x\leq \varepsilon \leq y\leq 1\\x\not= y}}\frac{\omega (f,y) ( \varepsilon -x ) +\omega (f,x) ( y-\varepsilon )}{y-x} , &0\leq \varepsilon \leq 1, \\ \omega (f,1), & \varepsilon >1. \end{cases} $$

### Theorem 4

*Suppose* $f\in C^{2} [ 0,1 ] $. *Then*

$$\begin{aligned}& \begin{aligned} &\biggl\vert G_{n}^{\tau } ( f;x ) -f ( x ) -\frac{1}{ \tau^{\prime } ( x ) } \biggl[ \frac{f^{\prime \prime } ( x ) \tau^{\prime } ( x ) -f^{\prime } ( x ) \tau^{\prime \prime } ( x ) }{ ( \tau^{\prime } ( x ) ) ^{2}} \biggr] \frac{\varphi_{n}^{2} ( x ) }{n+1}\biggr\vert \\ &\quad \leq \frac{\varphi_{n}^{2} ( x ) }{n+1}\tilde{\omega } \biggl( \bigl( f\circ \tau^{-1} \bigr) ^{\prime \prime };\frac{ \sqrt{6}}{3} ( n+3 ) ^{-1/2} \biggr) \end{aligned} \end{aligned}$$

*holds for every* $x\in [ 0,1 ] $.

### Proof

In Theorem 3, if we replace

$$ L_{n}f= \bigl( G_{n}^{\tau } \bigl( f\circ \tau^{-1} \bigr) \bigr) \circ \tau =G_{n}^{\tau }f $$

then we obtain

$$\begin{aligned}& \biggl\vert G_{n}^{\tau } ( f;x ) -\frac{1}{2} \bigl( f \circ \tau^{-1} \bigr) ^{\prime \prime } \bigl( \tau ( x ) \bigr) M_{n,2}^{\tau } ( x ) - \bigl( f\circ \tau^{-1} \bigr) ^{\prime } \bigl( \tau ( x ) \bigr) M_{n,1}^{\tau } ( x ) -f ( x ) \biggr\vert \\& \quad \leq \frac{M_{n,2}^{\tau } ( x ) }{2}\tilde{\omega } \biggl( \bigl( f\circ \tau^{-1} \bigr) ^{\prime \prime };\frac{1}{3}\sqrt{ \frac{M _{n,4}^{\tau } ( x ) }{M_{n,2}^{\tau } ( x ) }} \biggr) . \end{aligned}$$

Using (2.3) and (2.4) and the inequality (2.6), we immediately find that

$$\begin{aligned} \biggl\vert G_{n}^{\tau } ( f;x ) -f ( x ) - \bigl( f \circ \tau^{-1} \bigr) ^{\prime \prime } \bigl( \tau ( x ) \bigr) \frac{\varphi_{n}^{2} ( x ) }{n+1}\biggr\vert \leq \frac{ \varphi_{n}^{2} ( x ) }{n+1}\tilde{\omega } \biggl( \bigl( f \circ \tau^{-1} \bigr) ^{\prime \prime };\frac{\sqrt{6}}{3} ( n+3 ) ^{-1/2} \biggr) . \end{aligned}$$

Further, using (3.3), we obtain

$$\begin{aligned}& \biggl\vert G_{n}^{\tau } ( f;x ) -f ( x ) - \frac{1}{ \tau^{\prime } ( x ) } \biggl[ \frac{f^{\prime \prime } ( x ) \tau^{\prime } ( x ) -f^{\prime } ( x ) \tau^{\prime \prime } ( x ) }{ ( \tau^{\prime } ( x ) ) ^{2}} \biggr] \frac{\varphi_{n}^{2} ( x ) }{n+1}\biggr\vert \\& \quad \leq \frac{\varphi_{n}^{2} ( x ) }{n+1}\tilde{\omega } \biggl( \bigl( f\circ \tau^{-1} \bigr) ^{\prime \prime };\frac{ \sqrt{6}}{3} ( n+3 ) ^{-1/2} \biggr) , \end{aligned}$$

which completes the proof. □

### Corollary 1

*The following hold*:

(i) *Let* $f\in C^{2} [ 0,1 ] $. *If we choose* $\tau ( x ) =x$ *in Theorem* 4, *then we obtain a quantitative Voronovskaya theorem for the operators* $U_{n}$:
  $$ \biggl\vert U_{n} ( f;x ) -f ( x ) -f^{\prime \prime } ( x ) \frac{x ( 1-x ) }{n+1}\biggr\vert \leq \frac{x ( 1-x ) }{n+1}\tilde{\omega } \biggl( f^{\prime \prime };\frac{\sqrt{6}}{3} ( n+3 ) ^{-1/2} \biggr) . $$
(ii) *Let* $f\in C^{2} [ 0,1 ] $. *If we take a limit as* $n\rightarrow \infty $ *in Theorem* 4, *then we have the Voronovskaya theorem for* $G_{n}^{\tau }$:
  $$ \lim_{n\rightarrow \infty }n \bigl[ G_{n}^{\tau } ( f;x ) -f ( x ) \bigr] =\varphi_{n}^{2} ( x ) D^{2} \bigl( f \circ \tau^{-1} \bigr) \bigl( \tau ( x ) \bigr) . $$
(iii) *Let* $f\in C^{2} [ 0,1 ] $. *If* $n\rightarrow \infty $ *with* $\tau ( x ) =x$ *in the earlier Theorem* 4, *then we obtain the Voronovskaya theorem for* $U_{n}$:
  $$ \lim_{n\rightarrow \infty }n \bigl[ U_{n} ( f;x ) -f ( x ) \bigr] =x ( 1-x ) f^{\prime \prime } ( x ) . $$

The following result is a quantitative Grüss–Voronovskaya type theorem. For some applications of Grüss inequalities in approximation theory, one can refer to [22, 23].

### Theorem 5

*Let* $f\in C^{2} [ 0,1 ] $. *Then*, *for every* $x\in [ 0,1 ] $, *the inequality*

$$\begin{aligned}& \begin{aligned} &n\biggl\vert G_{n}^{\tau } ( fg;x ) -G_{n}^{\tau } ( f;x ) G_{n}^{\tau } ( g;x ) -\frac{2\varphi_{n}^{2} ( x ) }{ ( n+1 ) ( \tau^{\prime } ( x ) ) ^{2}} \biggl( g^{\prime } ( x ) f^{\prime } ( x ) -\frac{ ( fg ) ^{\prime } ( x ) \tau^{\prime \prime } ( x ) }{ ( \tau^{\prime } ( x ) ) } \biggr) \biggr\vert \\ &\quad \leq \frac{\varphi_{n}^{2} ( x ) }{n+1}\tilde{\omega } \biggl( \bigl( fg\circ \tau^{-1} \bigr) ^{\prime \prime };\frac{ \sqrt{6}}{3} ( n+3 ) ^{-1/2} \biggr) \\ &\quad \quad {}+\Vert f\Vert \frac{ \varphi_{n}^{2} ( x ) }{n+1}\tilde{\omega } \biggl( \bigl( g \circ \tau^{-1} \bigr) ^{\prime \prime };\frac{\sqrt{6}}{3} ( n+3 ) ^{-1/2} \biggr) \\ &\quad \quad {} +\Vert g\Vert \frac{\varphi_{n}^{2} ( x ) }{n+1}\tilde{\omega } \biggl( \bigl( f\circ \tau^{-1} \bigr) ^{\prime \prime };\frac{\sqrt{6}}{3} ( n+3 ) ^{-1/2} \biggr) +I_{n} ( f,x ) I_{n} ( g,x ) \end{aligned} \end{aligned}$$

*holds*.

### Proof

For all $x\in [ 0,1 ] $ and $n\in \mathbb{N}$, we can write

$$\begin{aligned}& G_{n}^{\tau } ( fg;x ) -G_{n}^{\tau } ( f;x ) G _{n}^{\tau } ( g;x ) -M_{n,2}^{\tau } ( x ) \frac{g ^{\prime } ( x ) f^{\prime } ( x ) }{ ( \tau^{\prime } ( x ) ) ^{2}} \\& \qquad {}-M_{n,2}^{\tau } ( x ) \frac{g ( x ) f^{\prime } ( x ) \tau^{\prime \prime } ( x ) }{ ( \tau^{\prime } ( x ) ) ^{3}}-M_{n,2}^{\tau } ( x ) \frac{g^{\prime } ( x ) f ( x ) \tau^{\prime \prime } ( x ) }{ ( \tau^{\prime } ( x ) ) ^{3}} \\& \quad = G_{n}^{\tau } ( fg;x ) -f ( x ) g ( x ) - \frac{M_{n,2}^{\tau } ( x ) }{2} \bigl( fg\circ \tau^{-1} \bigr) ^{\prime \prime } \bigl( \tau ( x ) \bigr) \\& \quad \quad {}-f ( x ) \biggl[ G_{n}^{\tau } ( g;x ) -g ( x ) - \frac{M_{n,2}^{\tau } ( x ) }{2} \bigl( g\circ \tau ^{-1} \bigr) ^{\prime \prime } \bigl( \tau ( x ) \bigr) \biggr] \\& \quad \quad {}-g ( x ) \biggl[ G_{n}^{\tau } ( f;x ) -f ( x ) - \frac{M_{n,2}^{\tau } ( x ) }{2} \bigl( f\circ \tau ^{-1} \bigr) ^{\prime \prime } \bigl( \tau ( x ) \bigr) \biggr] \\& \quad \quad {} + \bigl( g ( x ) -G_{n}^{\tau } ( g;x ) \bigr) \bigl( G_{n}^{\tau } ( f;x ) -f ( x ) \bigr) . \end{aligned}$$

From the equality (2.4), we have

$$\begin{aligned}& \biggl\vert G_{n}^{\tau } ( fg;x ) -G_{n}^{\tau } ( f;x ) G_{n}^{\tau } ( g;x ) -\frac{2\varphi_{n}^{2} ( x ) }{ ( n+1 ) ( \tau^{\prime } ( x ) ) ^{2}} \biggl( g^{\prime } ( x ) f^{\prime } ( x ) -\frac{ ( fg ) ^{\prime } ( x ) \tau^{\prime \prime } ( x ) }{ ( \tau^{\prime } ( x ) ) } \biggr) \biggr\vert \\& \quad \leq \vert \alpha_{1}\vert +\vert \alpha_{2} \vert + \vert \alpha_{3}\vert +\vert \alpha_{4} \vert . \end{aligned}$$

According to Theorem 4, we get

$$\begin{aligned}& \vert \alpha_{1}\vert \leq \frac{\varphi_{n}^{2} ( x ) }{n+1}\tilde{\omega } \biggl( \bigl( fg\circ \tau^{-1} \bigr) ^{\prime \prime }; \frac{\sqrt{6}}{3} ( n+3 ) ^{-1/2} \biggr) , \\& \vert \alpha_{2}\vert \leq \Vert f\Vert \frac{ \varphi_{n}^{2} ( x ) }{n+1} \tilde{\omega } \biggl( \bigl( g \circ \tau^{-1} \bigr) ^{\prime \prime }; \frac{\sqrt{6}}{3} ( n+3 ) ^{-1/2} \biggr) , \\& \vert \alpha_{3}\vert \leq \Vert g\Vert \frac{ \varphi_{n}^{2} ( x ) }{n+1} \tilde{\omega } \biggl( \bigl( f \circ \tau^{-1} \bigr) ^{\prime \prime }; \frac{\sqrt{6}}{3} ( n+3 ) ^{-1/2} \biggr) . \end{aligned}$$

On the other hand, by the assumptions of the theorem, we can write

$$ G_{n}^{\tau } ( f;x ) -f ( x ) = \bigl( f\circ \tau^{-1} \bigr) \bigl( \tau ( x ) \bigr) M_{n,1} ^{\tau } ( x ) +\frac{1}{2}G_{n}^{\tau } \bigl( \bigl( f \circ \tau^{-1} \bigr) ^{\prime \prime }\tau ( \xi ) \bigl( \tau ( t ) -\tau ( x ) \bigr) ^{2};x \bigr) $$

and using (2.3), we immediately find that

$$\begin{aligned} \bigl\vert G_{n}^{\tau } ( f;x ) -f ( x ) \bigr\vert & \leq \frac{1}{2}G_{n}^{\tau } \bigl( \bigl\vert \bigl( f\circ \tau^{-1} \bigr) ^{\prime \prime }\tau ( \xi ) \bigr\vert \bigl( \tau ( t ) -\tau ( x ) \bigr) ^{2};x \bigr) \\ &\leq \frac{1}{2}\bigl\Vert f\circ \tau^{-1}\bigr\Vert M_{n,2} ^{\tau } ( x ) \\ &=\frac{\varphi_{n}^{2} ( x ) \Vert f\circ \tau^{-1}\Vert }{n+1} \\ &:=I _{n} ( f,x ) . \end{aligned}$$

Therefore, we have

$$\begin{aligned}& n\biggl\vert G_{n}^{\tau } ( fg;x ) -G_{n}^{\tau } ( f;x ) G_{n}^{\tau } ( g;x ) -\frac{2\varphi_{n}^{2} ( x ) }{ ( n+1 ) ( \tau^{\prime } ( x ) ) ^{2}} \biggl( g^{\prime } ( x ) f^{\prime } ( x ) -\frac{ ( fg ) ^{\prime } ( x ) \tau^{\prime \prime } ( x ) }{ ( \tau^{\prime } ( x ) ) } \biggr) \biggr\vert \\& \quad \leq \frac{\varphi_{n}^{2} ( x ) }{n+1}\tilde{\omega } \biggl( \bigl( fg\circ \tau^{-1} \bigr) ^{\prime \prime };\frac{ \sqrt{6}}{3} ( n+3 ) ^{-1/2} \biggr) \\& \quad \quad {} +\Vert f\Vert \frac{ \varphi_{n}^{2} ( x ) }{n+1}\tilde{\omega } \biggl( \bigl( g \circ \tau^{-1} \bigr) ^{\prime \prime };\frac{\sqrt{6}}{3} ( n+3 ) ^{-1/2} \biggr) \\& \quad \quad {} +\Vert g\Vert \frac{\varphi_{n}^{2} ( x ) }{n+1}\tilde{\omega } \biggl( \bigl( f\circ \tau^{-1} \bigr) ^{\prime \prime };\frac{\sqrt{6}}{3} ( n+3 ) ^{-1/2} \biggr) +I_{n} ( f,x ) I_{n} ( g,x ) , \end{aligned}$$

which proves the theorem completely. □

The following corollary is a consequence of Theorem 5.

### Corollary 2

*One has the following*:

(i) *Let* $f\in C^{2} [ 0,1 ] $. *The choice of* $\tau ( x ) =x$ *in Theorem* 5 *gives a quantitative Grüss–Voronovskaya type theorem for* $U_{n}$:
  $$\begin{aligned}& n\biggl\vert U_{n} ( fg;x ) -U_{n} ( f;x ) U_{n} ( g;x ) -\frac{2x ( 1-x ) g^{\prime } ( x ) f^{\prime } ( x ) }{ ( n+1 ) }\biggr\vert \\& \quad \leq \frac{x ( 1-x ) }{n+1}\tilde{\omega } \biggl( ( fg ) ^{\prime \prime }; \frac{\sqrt{6}}{3} ( n+3 ) ^{-1/2} \biggr) + \frac{x ( 1-x ) \Vert f\Vert }{n+1} \tilde{ \omega } \biggl( g^{\prime \prime };\frac{\sqrt{6}}{3} ( n+3 ) ^{-1/2} \biggr) \\& \quad \quad {} +\frac{x ( 1-x ) \Vert g\Vert }{n+1} \tilde{\omega } \biggl( f^{\prime \prime }; \frac{\sqrt{6}}{3} ( n+3 ) ^{-1/2} \biggr) + \biggl( \frac{x ( 1-x ) }{n+1} \biggr) ^{2}\Vert f\Vert \Vert g\Vert . \end{aligned}$$
(ii) *Let* $f\in C^{2} [ 0,1 ] $. *If* $n\rightarrow \infty $ *in Theorem* 5, *we obtain the Grüss–Voronovskaya type theorem for* $G_{n}^{\tau }$:
  $$\begin{aligned}& \begin{aligned} &\lim_{n\rightarrow \infty }n \bigl[ G_{n}^{\tau } ( fg;x ) -G _{n}^{\tau } ( f;x ) G_{n}^{\tau } ( g;x ) \bigr] \\ &\quad =\frac{2 \varphi_{n}^{2} ( x ) }{ ( \tau^{\prime } ( x ) ) ^{2}} \biggl( g^{\prime } ( x ) f^{\prime } ( x ) - \frac{ ( fg ) ^{\prime } ( x ) \tau^{\prime \prime } ( x ) }{ ( \tau^{\prime } ( x ) ) } \biggr) . \end{aligned} \end{aligned}$$
(iii) *Let* $f\in C^{2} [ 0,1 ] $. *If* $n\rightarrow \infty $ *with* $\tau ( x ) =x$ *in Theorem* 5, *we obtain the Grüss–Voronovskaya type theorem for the operators* $U_{n}$:
  $$ \lim_{n\rightarrow \infty }n \bigl[ U_{n} ( fg;x ) -U_{n} ( f;x ) U_{n} ( g;x ) \bigr] =2x ( 1-x ) f^{\prime } ( x ) g^{\prime } ( x ) . $$

**Figure 1:**
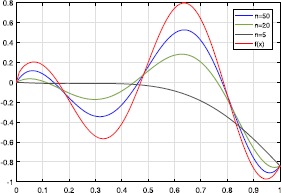
Approximation of $f(x)$ by $G_{n}^{\tau } ( f;x )$

We now present a graphic which shows the approximation of our new operators for the selection (see Fig. 1):

$$\begin{aligned}& f ( x ) = x^{1/2}\cos ( 10x ) , \\& \tau ( x ) = \bigl( x^{2}+2x \bigr) /3. \end{aligned}$$

### Remark 1

The further properties of the operators such as convergence properties via summability methods (see, for example, [24–26]) might be studied.

## Results and discussion

The results show that the new construction of the operators, that is, the genuine generalized Bernstein–Durrmeyer cases, are more effective in the approximation process than both the generalized Bernstein–Durrmeyer and the classical Bernstein–Durrmeyer operators. The other results are the quantitative form of Voronovskaya type results which present a new aspect to the pointwise approximation behavior of corresponding operators that we can use to investigate; the rate of pointwise convergence and an upper bound for the error of this pointwise approximation are presented simultaneously. As a point of discussion, another form of the operators than the King type can be studied and can be compared with these operators. Even a smaller error of approximation can be described by using a different modulus of continuity.

## Conclusion

In the paper, we constructed a new form of Bernstein–Durrmeyer operators, namely, genuine modified Bernstein–Durrmeyer operators. We have calculated the rate of convergence of our new operators by means of the modulus of smoothness. Also, the pointwise convergence properties of genuine modified Bernstein–Durrmeyer operators were discussed. Moreover, the significance of our results is supported by graphical and numerical data.
